# Impact of Age on Community Diabetes Prevention Program Attendance and Weight Loss Goals

**DOI:** 10.1093/geroni/igaa057.3328

**Published:** 2020-12-16

**Authors:** Jenna Napoleone, Elsa Strotmeyer, Rachel Miller, Susan Devaraj, Bonny Rockette-Wagner, Vincent Arena, Elizabeth Venditti, Andrea Kriska

**Affiliations:** University of Pittsburgh, Pittsburgh, Pennsylvania, United States

## Abstract

The Diabetes Prevention Program (DPP) lifestyle intervention demonstrated that meeting the weight loss (WL) and activity goals prevents/delays type 2 diabetes. Older DPP participants, 60-85 years, reduced the risk of developing diabetes by 71% versus 58% in those <60 years. Currently, community translated DPP-based lifestyle interventions including Group Lifestyle Balance (DPP-GLB), are reimbursed by Medicare for overweight/obese older adults with prediabetes. This effort examined the impact of age group (60-65: reference, 66-70, ≥71 years) on both DPP-GLB maintenance session attendance (months 7-12) and achieving the 5% WL goals at 6- and 12-months. Data were combined from two identical 12-month DPP-GLB intervention trials involving overweight/obese adults with prediabetes and/or metabolic syndrome. Participants ≥60 years attending ≥4 sessions (months 0-6), with complete data on session attendance and WL were included (n=145; age=68.7 + 5.8 years, range 60-88; 79% women). Participants aged 66-70 years (N=46) were more likely to meet the 6-month 5% WL goal (67.4%) vs. 60-65 years (N=51; 45.1%; p=0.03). Participants aged 66-70 (69.6%) and ≥71 years (N=48; 60.4%) were more likely to meet the 12-month WL goal vs. 60-65 years (35.3%; 66-70: p=0.0007; ≥71: p=0.01). Maintenance attendance did not vary by age group with approximately 30% of each group attending ≥4 of 6 maintenance sessions (p=0.55). In conclusion, adults 66+ vs. 60-65 years more successfully met the clinically meaningful 5% WL goals at 6 and 12 months. With nationwide implementation of community-based “real-world” DPP-GLB lifestyle interventions, better understanding of program success across older adult age groups will enhance program reach and effectiveness.

